# IRF8 Impacts Self‐Renewal of Hematopoietic Stem Cells by Regulating TLR9 Signaling Pathway of Innate Immune Cells

**DOI:** 10.1002/advs.202101031

**Published:** 2021-08-08

**Authors:** Donghe Li, Yuyin Zhang, Qingsong Qiu, Jinzeng Wang, Xuemei Zhao, Bo Jiao, Xiuli Zhang, Shanhe Yu, Pengfei Xu, Yuqing Dan, Xinhua Xiao, Peihong Wang, Mingzhu Liu, Zhizhou Xia, Zhangsen Huang, Ruihong Zhang, Jiaoyang Li, Xi Xie, Yan Zhang, Chenxuan Liu, Ping Liu, Ruibao Ren

**Affiliations:** ^1^ Shanghai Institute of Hematology State Key Laboratory for Medical Genomics National Research Center for Translational Medicine International Center for Aging and Cancer Collaborative Innovation Center of Hematology Ruijin Hospital affiliated to Shanghai Jiao Tong University School of Medicine School of Life Sciences and Biotechnology Shanghai Jiao Tong University Shanghai 200025 China

**Keywords:** hematopoietic stem cells, IRF8, natural killer cells, proinflammatory cytokines, TLR9

## Abstract

IRF8 is a key regulator of innate immunity receptor signaling and plays diverse functions in the development of hematopoietic cells. The effects of IRF8 on hematopoietic stem cells (HSCs) are still unknown. Here, it is demonstrated that IRF8 deficiency results in a decreased number of long‐term HSCs (LT‐HSCs) in mice. However, the repopulation capacity of individual HSCs is significantly increased. Transcriptomic analysis shows that IFN‐*γ* and IFN‐*α* signaling is downregulated in IRF8‐deficient HSCs, while their response to proinflammatory cytokines is unchanged ex vivo. Further tests show that *Irf8* ^−/−^ HSCs can not respond to CpG, an agonist of Toll‐like receptor 9 (TLR9) in mice, while long‐term CpG stimulation increases wild‐type HSC abundance and decreases their bone marrow colony‐forming capacity. Mechanistically, as the primary producer of proinflammatory cytokines in response to CpG stimulation, dendritic cells has a blocked TLR9 signaling due to developmental defect in *Irf8* ^−/−^ mice. Macrophages remain functionally intact but severely reduce in *Irf8* ^−/−^ mice. In NK cells, IRF8 directly regulates the expression of *Tlr9* and its deficiency leads to no increased IFN*γ* production upon CpG stimulation. These results indicate that IRF8 regulates HSCs, at least in part, through controlling TLR9 signaling in diverse innate immune cells.

## Graphical abstract

This study reveals the effects of IRF8 function on hematopoietic stem cells (HSCs). *Irf8* ^−/−^ mice exhibit a decrease number yet enhanced repopulation capacity of HSCs. Mechanistically, IRF8 affects the release of proinflammatory cytokines by controlling the development of monocytes/macrophages and dendritic cells and the expression of TLR9 in NK cells. Consequently, the cytokine‐mediated HSC proliferation and exhaustion are reduced.

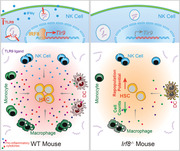

## 1 Introduction

Hematopoietic stem cells are located at the apex of the hematopoiesis hierarchy and are responsible for life‐long production of blood and immune cells.^[^ ^1^, ^2^ ^]^ HSCs require a special microenvironment, called “niche,” for proper self‐renewal and differentiation into progenitor cells. Most HSCs remain in a dormant state to preserve their capacity for self‐renewal, but in the case of infection, severe bleeding, or irradiation and chemotherapy, they can promptly proliferate and differentiate in response to cellular progeny lost.^[^ ^1^, ^3^, ^4^, ^5^ ^]^

Recent findings have highlighted that similar to immune effector cells, HSCs can also respond to inflammatory cytokines, chemokines, and TLR agonists, all of which can induce expansion, differentiation and migration. Generally, as a response to replenish immune cells, this reaction of HSCs to the inflammatory stimuli can be divided into two distinct sensing mechanisms: direct and indirect.^[^ ^1^, ^2^ ^]^ In the direct sensing mechanism, HSCs themselves recognize inflammatory cytokines or microbial products via receptors for cytokines and pattern recognition receptors (PRRs), such as TLRs, expressed on the surface of HSCs. Classically, type I and type II interferons, IL‐1*β*, IL‐6, M‐CSF (macrophage‐colony stimulating factor) and LPS (lipopolysaccharide) have been considered as the main direct activators of HSCs.^[^ ^6^, ^7^, ^8^ ^]^ Alternatively, HSCs can be activated indirectly, in which, exogenous stimuli cannot active HSCs directly, but mediated by mature immune cells that sense antigen and produce cytokines, such as IL‐1*β*, IFN*γ*, GM‐CSF, and type I and II IFN, that eventually stimulate HSCs. Poly(I:C), a synthesized double strand RNA acts as TLR3‐specific agonist, and *M. avium* infection, stimulates HSCs to proliferate in an IFN*α*‐ and IFN*γ*‐dependent fashion, respectively.^[^ ^8^, ^9^ ^]^ Such responses to infection by HSCs can be beneficial for promoting pathogen clearance. However, increasing evidence suggests that they may also lead to HSCs exhaustion and functional impairment.

Interferon regulatory factor 8 (IRF8), also known as IFN consensus sequence binding protein (ICSBP), is a key transcription factor expressed almost exclusively in hematopoietic cells which has been extensively studied in hematopoietic system including multi‐, oligo‐, and committed progenitors, as well as in immature and mature blood and immune cells. IRF8 acts in the lineage‐committed progenitors to selectively limit neutrophil production and promote monocyte production. Therefore, IRF8‐null mice have elevated neutrophil counts and reduced numbers of monocytes in both bone marrow and peripheral blood.^[^ ^10^ ^]^ IRF8 also plays a crucial role in dendritic cell differentiation, the deficiency of which leads to depletion of conventional CD8^+^ DC1s (cDC1s) and development of abnormal plasmacytoid dendritic cells (pDCs).^[^ ^11^, ^12^ ^]^ In addition, although IRF8 is not expressed in basophils, mast cells or basophil/mast cells progenitors (BMCPs), *Irf8^−/−^* mice display a severe reduction in basophil and mast cells. This phenomenon is mainly due to IRF8 deficiency in granulocyte progenitors (GPs), which are upstream of BMCPs and are unable to efficiently generate both cell lineages.^[^ ^13^ ^]^ In line with these phenotypes, *Irf8* gene deletion in mice results in a myeloproliferative syndrome and highly susceptible to infection by a variety of pathogens.

Due to its low expression levels in HSCs, the functions of IRF8 in these cells remain unclear.^[^ ^14^ ^]^ In this study, we investigated the impact of IRF8 upon HSCs and explored its underlying mechanisms.

## 2 Results

### 2.1 IRF8‐Deficient Mice Exhibit A Reduction in Long‐Term HSCs

**Figure 1:**
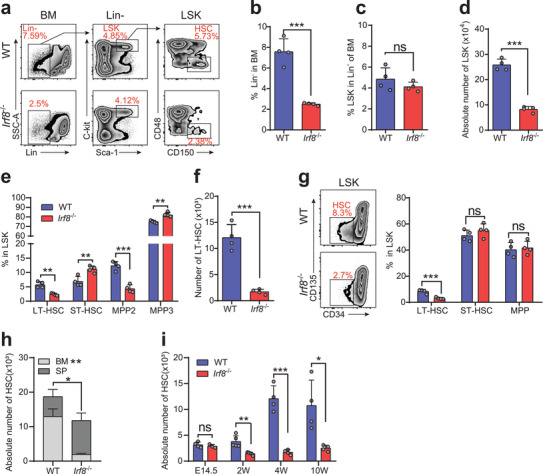
Loss of IRF8 reduces the number of HSC in mice after birth. a) Representative FACS plots of Lin^−^ cells, LSKs, and CD48^−^ CD150^+^ LT‐HSCs (SLAM family markers for HSCs) from BM of 4‐week‐old WT and *Irf8* knockout (*Irf8* ^−/−^) mice. b) Percentage of lineage negative cells in total BM and c) percentage of LSK (Lin^−^ Sca‐1^+^ c‐kit^+^) cells in total Lin^−^ cells. d) Absolute numbers of LSKs in 4‐week‐old WT and *Irf8* ^−/−^ mice. e) Proportions of different SLAM‐defined populations in the LSK compartment. LT‐HSC (LSK, CD48^−^ CD150^+^), ST‐HSC (LSK, CD48^−^ CD150^−^), MPP2 (LSK, CD48^+^ CD150^+^), MPP3 (LSK, CD48^+^ CD150^−^). f) Absolute numbers of SLAM‐defined LT‐HSCs in 4‐week‐old WT and *Irf8* ^−/−^ mice. g) Representative FACS plots of CD34‐ and CD135‐ defined LT‐HSCs (left) and proportions of different subpopulations in the LSK compartment (right). h) Total numbers of LT‐HSCs from BM and spleen of 4‐week‐old WT and *Irf8* ^−/−^ mice. i) Absolute numbers of LT‐HSCs (SLAM‐defined) from fetal liver or BM of mice at different ages. Error bars, mean ± s.e.m. ns, no significance, *P < 0.05, **P < 0.01, ***P < 0.001, data representing two or more independent experiments were analyzed with unpaired Student's *t*‐test.

Although previous studies have shown that the expression of IRF8 is relatively low in HSCs,^[^ ^14^, ^15^, ^16^ ^]^ the possibility of IRF8 function in HSCs cannot be ruled out. To explore the intrinsic effects of IRF8 on HSCs and to avoid the effects of myeloproliferative disorder syndrome (MPD) occurring in *Irf8^−/−^* mice with age,^[^ ^17^ ^]^ young mice (4–6‐week‐old) were used in this study. Analysis of bone marrow cells (BM) revealed a reduction in the absolute cell numbers in the LSK (Lin*^−^* Sca‐1^+^c‐Kit^+^) compartment, which mainly resulted from the decreased proportion of lineage‐negative cells (Lin*^−^*), but the ratio of LSKs to all Lin*^−^* cells was unchanged (**Figure** **1a**–d). Despite a slight increase in BM cells in *Irf8^−/−^* mice, this could not make up for the nearly three‐fold shortfall in Lin*^−^* cells (Figure 1d). Further results showed that, compared to wild‐type (WT) mice, loss of IRF8 significantly reduced the frequency and total number of SLAM (signaling lymphocytic activation molecule)‐defined LT‐HSCs (LSK, CD48*^−^* CD150^+^),^[^ ^18^ ^]^ as well as multipotent progenitors (MPP2, LSK, CD48^+^CD150^+^) (Figure 1e,f). In contrast, the proportions of ST‐HSCs (short‐term HSC: LSK, CD48*^−^* CD150*^−^*) and MPP3 (LSK, CD48^+^CD150*^−^*) cells were significantly increased (Figure 1e). Given that CD150^+^ subpopulations decreased in IRF8‐deficient mice, we must exclude the possibility that phenotypic HSCs were incorrectly identified as a consequence of abnormal expression of a single HSC‐defined marker. Therefore, we assessed the proportions of LT‐HSCs by another staining scheme, LSK, CD34*^−^* CD135*^−^*, and the results were consistent with that of SLAM‐defined LT‐HSCs (Figure 1g).

In the spleen, we found that the proportion of *Irf8^−/−^* LKs (Lin*^−^* c‐Kit^+^) was significantly increased, while the proportion of LT‐HSCs in LSKs was decreased (Figure S1a–c, Supporting Information). Although the absolute numbers of LKs and LT‐HSCs were increased due to splenomegaly (Figure S1d, Supporting Information), the sum of HSCs in bone marrow and spleen was still significantly reduced in *Irf8^−/−^* mice (Figure 1h). Collectively, these results showed that IRF8‐deficient mice, at the age of 4–6 weeks, had a significantly decreased proportion of HSCs in LSKs and lower total HSC numbers compared to WT mice.

During ontogeny, the entire HSC population undergoes cycling until 3 weeks after birth in mice, after which the majority of these cells switch to a quiescent state.^[^ ^19^ ^]^ To further investigate which stages of ontogeny are affected by IRF8 in HSCs, fetal liver of E14.5 and BM of 2, 4, 10 weeks after birth were measured. We found that the cell counts in fetal liver, the ratio of LSKs in Lin*^−^* cells, and the percentage of HSCs in LSKs in E14.5 were not significantly different between *Irf8^−/−^* and WT mice (Figure S1e–i, Supporting Information). In addition, HSCs decreased starting at 2 weeks after birth in IRF8‐deficent mice, and this gap widened further in the 4th week and remained so through the 10th week (Figure 1i).

To explore the possible mechanisms driving HSCs reduction in *Irf8^−/−^* mice, we examined the distribution of cells at different phases of the cell cycle, proliferation, and apoptosis. Hoechst 33 342/Ki‐67 staining showed no difference in cell cycle status between *Irf8^−/−^* and WT LT‐HSCs, although the former had a slight, but nonsignificant, increase in the proportion of S/G2/M phases (Figure S2a,b, Supporting Information). Consistent with this finding, BrdU (bromodeoxyuridine) incorporation assays showed that the proliferation of *Irf8^−/−^* LT‐HSCs decreased marginally (Figure S2c,d, Supporting Information). In addition, no increase in apoptosis was detected among HSCs with IRF8 deficiency (Figure S2e, Supporting Information).

### 2.2 *Irf8^−/−^* LT‐HSCs Have an Enhanced Capacity of Self‐Renewal and Reconstitution

**Figure 2:**
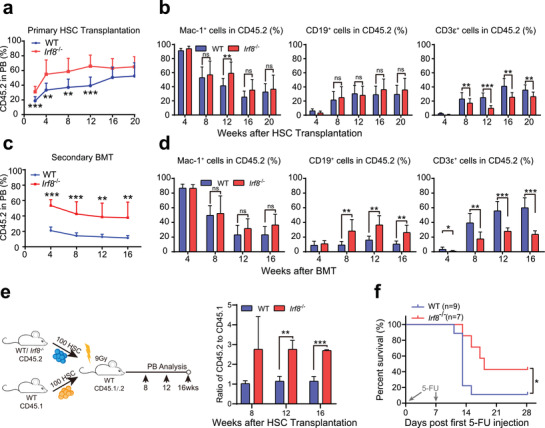
IRF8 knockout enhanced the self‐renewal capacity of individual LT‐HSCs. a) Long‐term follow‐up of donor‐derived cell (CD45.2^+^ cells) proportions in peripheral blood (PB) of primary HSC transplantation mice (WT: *n* = 10; *Irf8* ^−/−^: *n* = 10). b) Ratio of Mac‐1^+^ myeloid cells, CD19^+^ B cells, and CD3*ε* ^+^ T cells to the total donor‐derived cells in PB from 4 to 20 weeks after primary HSC transplantation (WT: *n* = 10; *Irf8* ^−/−^: *n* = 10). c) Long‐term follow‐up of donor‐derived cell proportions in PB of secondary BM transplantation; and d) ratio of various lineages of donor‐derived cell in PB (WT: *n* = 7; *Irf8* ^−/−^: n = 9). e) Experimental scheme for competitive HSC transplantation (left panel), normalized ratio of CD45.2^+^ over CD45.1^+^ cells in PB of competitive BM transplanted mice (WT: *n* = 3; *Irf8* ^−/−^: *n* = 4) (right panel). f) Kaplan–Meier survival curves of WT and *Irf8* ^−/−^ mice treated twice with 5‐FU. Error bars, mean±s.e.m. *P < 0.05, **P < 0.01, ***P < 0.001, data were analyzed with unpaired Student's *t*‐test.

The reduction of phenotypical LT‐HSCs prompted us to investigate whether the function of individual HSCs was affected in *Irf8^−/−^* mice. To this end, we first performed a transplantation assay to evaluate the capacity for self‐renewal and multipotent differentiation ability of *Irf8^−/−^* LT‐HSCs. LT‐HSCs harvested from WT or *Irf8^−/−^* BM of mice with a CD45.2 genetic background were sorted and transplanted into CD45.1 recipients, and the donor‐derived cells in PB were monitored every 4 weeks. The results showed that the percentages of donor‐derived CD45.2 cells in *Irf8^−/−^* transplants were significantly higher than those in WT transplants (**Figure** **2a**). Notably, despite a slight increase of peripheral white blood cell counts and splenomegaly in the recipient mice transplanted with *Irf8^−/−^* HSCs (Figure S3a,b, Supporting Information), they did not exhibit any clear myeloid bias (Figure 2b), which was consistent with previous research.^[^ ^20^ ^]^

To assess the self‐renewal capacity of *Irf8^−/−^* LT‐HSCs, a secondary transplantation was performed. In this experiment, 1 × 10^6^ BM cells from WT and *Irf8^−/−^* recipients of primary transplantation were transplanted into CD45.1 genetic background mice. The results revealed that *Irf8^−/−^* mice had an increased capacity for hematopoietic reconstitution (Figure 2c), including significant improvement in B cell repopulation and a slight increase in myeloid‐lineage differentiation, as well as a significantly lower proportion of T cells compared with WT mice (Figure 2d). Interestingly, splenomegaly was not observed in recipient mice of *Irf8^−/−^* LT‐HSCs at the final analysis 16 weeks after transplantation (Figure S3c, Supporting Information).

To further confirm these results, we performed a competitive HSC transplantation assay. Purified LT‐HSCs from WT or *Irf8^−/−^* BM (CD45.2 genetic background) were mixed with an equal number of CD45.1 HSCs and then transplanted into CD45.1/.2 recipient mice (Figure 2e). In agreement with our previous finding, *Irf8^−/−^* HSCs also showed improvement in hematopoietic reconstitution during the follow‐up period for 16 weeks (Figure 2e). Taken together, these results suggested that, although loss of IRF8 led to a marked decrease in LT‐HSCs, their self‐renewal and capacity for competitive repopulation were enhanced in individual LT‐HSC.

To analyze the systemic impact of IRF8 deficiency in hematopoiesis, WT and *Irf8^−^* ^/^ *^−^* mice were injected two times with a sublethal dose of 5‐FU (5‐Fluorouracil), which induces HSC exhaustion and bone marrow failure. A significant delay of 5‐FU‐induced hematopoietic failure was observed in *Irf8^−^* ^/^ *^−^* mice (Figure 2f), confirming the conclusion that loss of IRF8 enhanced HSC self‐renewal.

### 2.3 Knockout of *Irf8* Impairs the Response of HSPCs to TLR9 Agonists In Vivo

**Figure 3:**
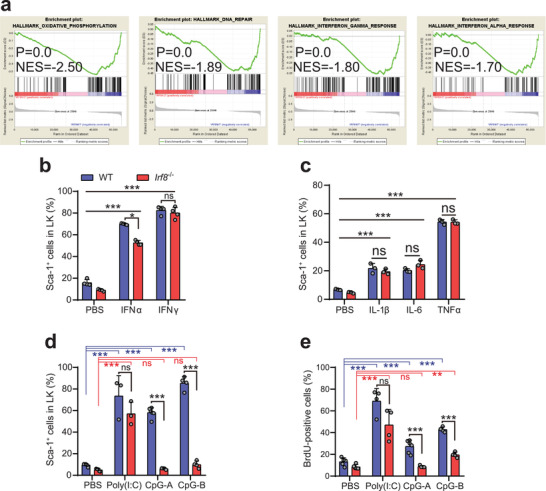
Loss of IRF8 impair the activation of HSCs by TLR9 agonists in vivo. a) Gene set enrichment analysis of RNA‐Seq data of LT‐HSCs isolated from 4‐week‐old WT and *Irf8* ^−/−^ mice at steady‐state (WT: *n* = 3; *Irf8* ^−/−^: *n* = 3). Gene sets of “oxidative phosphorylation,” “DNA repair,” “interferon gamma response,” and “interferon alpha response” were enriched. b) Sca‐1 expression of purified LKs isolated from WT and *Irf8* ^−/−^ mice that were stimulated or not with IFN‐*α*(100 ng mL^−1^) or IFN‐*γ* (100 ng mL^−1^) ex vivo for 20 h. c) Proportions of Sca‐1‐positive cells in purified WT and *Irf8* ^−/−^ LKs at 16 h after stimulation with various cytokines ex vivo. d) Sca‐1 expression in LKs and e) 16 h BrdU incorporation in LT‐HSCs from BM of WT and *Irf8* ^−/−^ mice stimulated or unstimulated with two injections of Poly(I:C) (i.p.) (for 72 h) or by single injection of CpG‐A or CpG‐B (i.v.) (for 24 h). Error bars, mean ± s.e.m. ns, no significance, *P < 0.01, ***P < 0.001, data representing two or more independent experiments were analyzed with unpaired Student's *t*‐test.

To further explore the characteristic of HSCs in IRF8‐deficient mice, we next performed RNA sequencing (RNA‐seq) analysis using LT‐HSCs from 4‐week‐old WT and *Irf8^−/−^* mice. Consistent with the observed increase in self‐renewal capacity, gene sets associated with cell metabolism and DNA repair were down‐regulated in IRF8‐deficient LT‐HSCs (**Figure** **3a**). More notably, interferon gamma and alpha response pathways were down‐regulated in *Irf8^−/−^* LT‐HSCs (Figure 3a), which prompted us to test the response of *Irf8^−/−^* HSCs exposed to IFN‐*γ* or IFN‐*α*. To this end, purified WT and *Irf8^−/−^* LKs were treated with IFN‐*α* or IFN‐*γ*, and the expression of Sca‐1, an upregulated marker on HSPCs (hematopoietic stem and progenitor cells) under acute inflammation,^[^ ^8^, ^9^ ^]^ was measured. Expression of Sca‐1 in LKs isolated from both WT and *Irf8^−/−^* BM was significantly up‐regulated in response to IFN‐*γ* or IFN‐*α* (Figure 3b). In addition, other pro‐inflammatory cytokines including TNF‐*α*, IL‐1‐*β*, and IL‐6 were tested ex vivo and the same effects were observed between WT LKs and *Irf8^−/−^* LKs. (Figure 3c). Together these data indicated that *Irf8^−^* ^/^ *^−^* HSPCs had no significant differences from WT HSPCs in response to pro‐inflammatory cytokines.

Extensive studies have revealed that several members of the IRF family, e.g., IRF3, IRF5, and IRF7, are involved in TLR signaling.^[^ ^21^, ^22^, ^23^ ^]^ In addition, IRF8 has been associated with TLR signaling,^[^ ^24^ ^]^ especially the TLR9 pathway in pDCs.^[^ ^11^, ^25^ ^]^ Therefore, we hypothesized that the phenotypical and functional changes of HSCs in *Irf8^−/−^* mice were due to impaired TLR signaling. To test this hypothesis, WT and *Irf8^−/−^* mice were treated with different TLR agonists and Sca‐1 expression and BrdU incorporation were assessed in LKs and LT‐HSCs, respectively. After treatment with Poly(I:C), a strong type I IFN inducer and TLR3 agonist, we found the Sca‐1 expression was markedly induced in both WT and *Irf8^−/−^* BM‐derived LKs (Figure 3d). Similarly, BrdU incorporation by LT‐HSCs showed a significant increase following Poly(I:C) stimulation, independent of IRF8 deficiency (Figure 3e). In contrast, the Sca‐1 expression by *Irf8^−/−^* LKs and BrdU incorporation of LT‐HSCs were significantly lower than that of WT at 24 h after intravenous injection with TLR9 agonists, class A CpG (CpG‐A), or class B CpG (CpG‐B) (Figure 3d,e).

These data demonstrated that the response of HSPCs to TLR9 agonists, but not TLR3 agonist, was severely impaired in *Irf8^−/−^* mice, and IRF8 was essential for HSC response to TLR9 signaling.

### 2.4 TLR9 Agonists Stimulate Mouse HSPCs in an Indirect Manner

**Figure 4:**
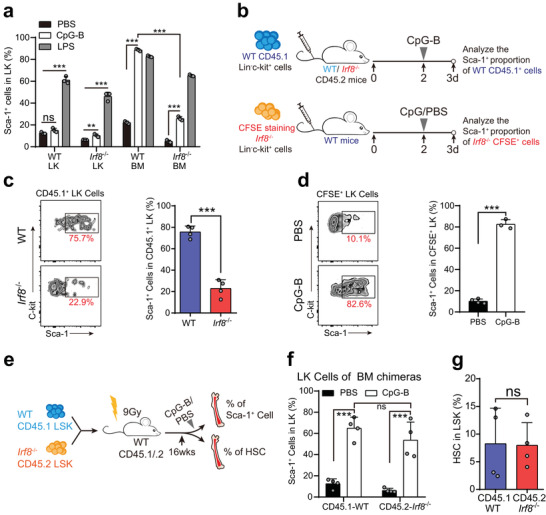
An indirect mechanism promotes HSPC activation in response to CpG. a) The proportions of Sca‐1 positive cells of LKs in BM cells or purified LKs stimulated or unstimulated with CpG‐B (200 ng mL^−1^) or LPS (100 EU mL^−1^) ex vivo. b) Experimental scheme for c) and d). In upper panel, purified WT LKs (CD45.1) were transferred into WT or *Irf8* ^−^ *^/^* ^−^ mice (CD45.2) and injected CpG‐B after 2 days. BM cells were analyzed on the third day. In lower panel, CFSE‐stained *Irf8* ^−^ *^/^* ^−^ LKs were adoptively transferred into WT recipient mice. Two days later recipients were treated with PBS or CpG‐B. BM cells were harvested three days after transplantation. c) Representative flow plots of the percentage of Sca‐1‐positive cells in donor‐derived WT LKs (CD45.1^+^ cells) after CpG‐B challenge and histograms of the statistical quantification. d) The percentages of Sca‐1‐positive cells in donor‐derived CFSE^+^ LKs stimulated or unstimulated with CpG‐B. e) Establishment of bone marrow chimeras. f) Lethally irradiated WT (CD45.1/.2) mice were reconstituted with WT (CD45.1) and *Irf8* ^−^ *^/^* ^−^ (CD45.2) LSKs. At four months later, BM chimeric mice were treated with PBS or CpG‐B for 24 h and Sca‐1 expression in donor‐derived LKs was analyzed with flow cytometry. g) The proportions of HSCs in WT and *Irf8* ^−^ *^/^* ^−^ LSKs were analyzed In BM chimeric mice. Error bars, mean ± s.e.m. ns, no significance, **P < 0.01, ***P <0 .001, data representing two or more independent experiments were analyzed with unpaired Student's *t*‐test.

IRF8 plays a crucial role in HSPC response to CpG, which could be explained by two possible mechanisms: i) HSPCs directly sense CpG via TLR9 expressed on its intracellular membrane and this response is IRF8 dependent, or ii) CpG stimulates mature immune cells to secrete proinflammatory factors that activate HSPCs to proliferate via their specific receptors. To address this question, we first treated purified LKs directly with CpG or LPS for 16–20 h and found that neither WT nor *Irf8^−^* ^/^ *^−^* LKs showed a significant increase in their Sca‐1 expression under CpG treatment (**Figure** **4a**). We next performed the same assay to investigate the response of LKs to CpG in a whole BM environment, and found that the LKs cultured in WT BM had significantly higher Sca‐1 expression than those in *Irf8^−/−^* BM. As a positive control for direct activation, all LPS‐treated groups exhibited remarkably enhanced levels of Sca‐1 expression (Figure 4a). These results suggested that, unlike LPS, CpG appeared to activate HSPCs in a bone marrow microenvironment‐dependent manner.

To confirm our observations in vivo, we transplanted WT LKs (CD45.1 genetic background) into unirradiated WT and *Irf8^−/−^* mice (both CD45.2 background), and stimulated the recipients with CpG two days after transplantation (Figure 4b). As expected, the donor‐derived LKs increased their Sca‐1 expression after CpG stimulation only in the WT BM environment (Figure 4c). In another test, we transplanted *Irf8^−/−^* LKs labeled with CFSE (carboxyfluorescein succinimidyl ester) into unirradiated WT recipients prior to CpG administration (Figure 4b). Similar to WT LKs, *Irf8^−/−^* LKs showed remarkably improved Sca‐1 expression in the WT BM environment (Figure 4d). Thus, the response of HSPCs to CpG was in a BM environment dependent manner, which differed from their direct response to LPS.

### 2.5 Long‐Term Stimulation with CpG Affects the Number of HSCs

Based on the above results, we hypothesized that a normal immune environment could recover the proportion of *Irf8* ^−/−^ HSCs in LSKs. To investigate this possibility, we generated BM chimeric mice by reconstituting lethally irradiated WT mice (CD45.1/.2) with a mixture of WT (CD45.1) and *Irf8* ^−^ *^/^* ^−^ (CD45.2) LSKs (Figure 4e). After 4 months of reconstitution, the recipient mice were injected intravenously with PBS (phosphate‐buffered saline) or CpG. Again, we observed no differences between *Irf8* ^−/−^ and WT LKs in response to CpG in the chimeric hematopoietic environment (Figure 4f). Furthermore, in chimeric mice, we compared the proportions of HSCs in LSKs of WT and *Irf8* ^−/−^, and found that there was no significant difference (Figure 4g). These results suggested that the proportion of *Irf8* ^−/−^ HSCs in the LSK compartment was rescued by a normal immune environment.

To verify the role of the TLR9 signaling pathway in regulating the abundance and function of HSCs, we administered eight successive doses of CpG‐B or PBS in WT mice (Figure S4a, Supporting Information). When hematopoiesis returned to a balanced steady‐state (at one week after the last injection), the proportions and the number of HSCs were measured (Figure S4b, Supporting Information). Supporting our hypothesis, long‐term activation of TLR9 signaling led to an increase in both the absolute number of HSCs and their proportion in the LSK subpopulation (Figure S4b,c, Supporting Information). We next performed colony‐forming unit (CFU) assays to evaluate the functional effects of long‐term CpG‐B stimulation on BM hematopoietic cells. Compared with the PBS controls, CpG‐B‐treated BM cells produced fewer colonies in methylcellulose medium (Figure S4d, Supporting Information). Taken together, these results suggested that long‐term activation of TLR9 signaling affected the quantity and function of HSCs.

### 2.6 Aberrantly Low Levels of Proinflammatory Cytokines in Bone Marrow of *Irf8* ^−^ *^/^* ^−^ Mice Upon CpG Stimulation

**Figure 5:**
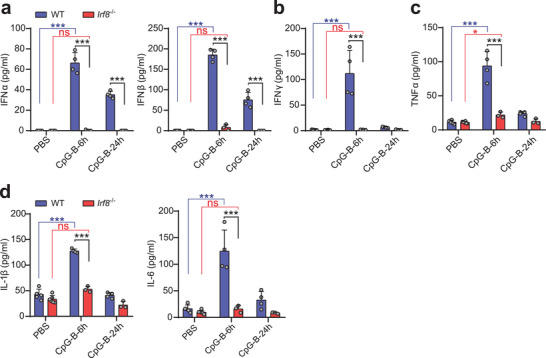
The deficiency of proinflammatory cytokines is the immediate cause for the abolished CpG response by HSC in *Irf8* ^−^ *^/^* ^−^ mice. a) The levels of type I interferons, b) type II interferons, c) TNF‐*α*, d) IL‐1*β* and IL‐6 in bone marrow cavity at 6 and 24 h after CpG‐B stimulation in vivo. Error bars, mean ± s.e.m. ns, no significance, ***P < 0.001, data were analyzed with unpaired Student's *t*‐test.

TLR9 recognizes DNA derived from both DNA viruses and bacteria and can promote the production of proinflammatory cytokines by effector immune cells,^[^ ^22^ ^]^ in which interferons and multiple cytokines have been shown to directly mobilize mouse HSCs.^[^ ^2^ ^]^ To investigate whether the impaired response of HSCs to TLR9 agonists in the *Irf8* ^−^ *^/^* ^−^ environment was due to aberrant proinflammatory cytokines production, we analyzed the levels of pro‐inflammatory cytokines in WT and *Irf8* ^−^ *^/^* ^−^ bone marrow cavities at 6 and 24 h after CpG treatment (**Figure** **5a**–d). We found that type I interferons (IFN‐*α* and IFN‐*β*), type II interferons (IFN‐*γ*), TNF*α*, IL‐1*β*, and IL‐6 in the bone marrow cavity all increased significantly at 6 h after injection, then decreased by 24 h in WT mice (Figure 5a–d). However, in *Irf8* ^−^ *^/^* ^−^ mice, these cytokines were only slightly elevated or even unchanged within 24 h after CpG treatment (Figure 5a–d). These data demonstrated that, *Irf8* ^−^ *^/^* ^−^ mice could not produce sufficient interferons and other proinflammatory cytokines in BM, driving an impaired response by HSCs to CpG.

### 2.7 The Developmental Defect of Primary CpG‐Responsive Cells in *Irf8* ^−/−^ Mice Disables Proinflammatory Cytokine Production

**Figure 6:**
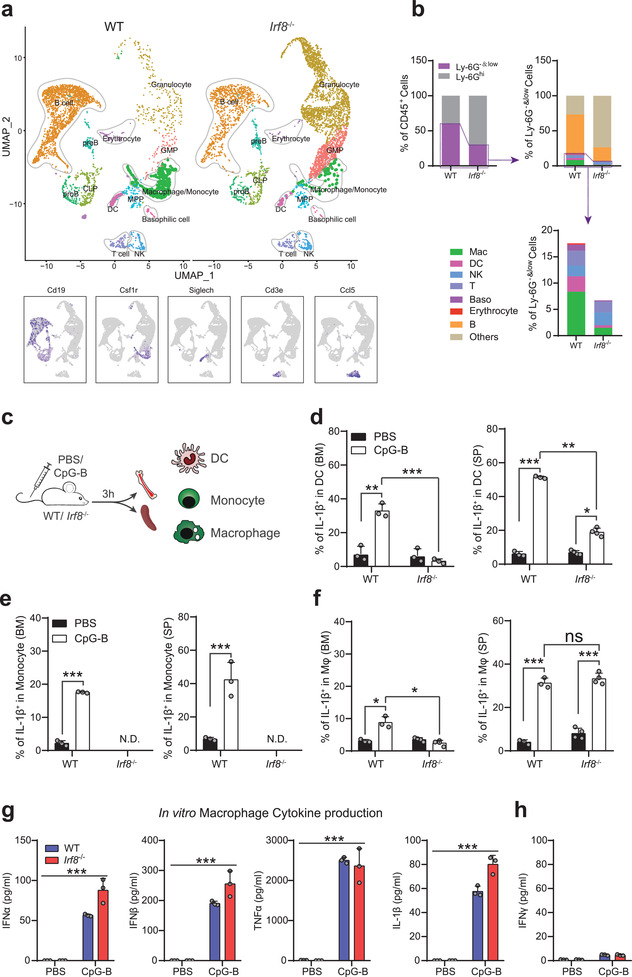
Loss of IRF8 affects the activation of TLR9 signaling in IRF8‐dependent immune cells under CpG‐B stimulation. a) UMAP (uniform manifold approximation and projection) plot of color‐coded clusters in Ly‐6G^hi^ depletion BM cells from WT and *Irf8* ^−^ *^/^* ^−^ mice. Dashed lines generally encompass immune cells and decreased subpopulations in *Irf8* ^−^ *^/^* ^−^ Ly‐6G^−&low^ cells. b) The proportions of Ly‐6G^hi^ and Ly‐6G^−&low^ cells in WT and *Irf8* ^−^ *^/^* ^−^ BM (top left panel), and cluster ratios of cells from single‐cell sequencing (right top and bottom panels). c) WT and *Irf8* ^−^ *^/^* ^−^ mice were treated with PBS or CpG‐B i.v. for 3 h and the IL‐1*β* ^+^ cells were measured in immune cells highly expressing TLR9 by flow cytometry. Proportions of IL‐1*β* ^+^ cells to d) BM cDCs (left) and spleen cDCs (right), e) BM monocytes (left) and spleen monocytes (right) as well as f) BM macrophages (left) and spleen macrophages (right). g) IFN‐*α*, IFN‐*β*, TNF‐*α*, IL‐1*β* and h) IFN‐*γ* production by WT and *Irf8* ^−^ *^/^* ^−^ BMDMs at 24 h after PBS or CpG‐B stimulation. Error bars, mean ± s.e.m. N.D., not detected, ***P < 0.001, data were analyzed with unpaired Student's *t*‐test.

CpG can directly activates pDCs, cDCs, macrophages/monocytes, and B cells via TLR9, while IRF8 is essential for the development of all of these subpopulations. Therefore, we assessed the impact of IRF8 deficiency by single‐cell transcriptomic profiling of purified BM cells following Ly‐6G^hi^ granulocyte depletion by fluorescence‐activated cell sorting (FACS) (Figure S5a, Supporting Information). Notably, the proportions of Ly‐6G^hi^ granulocytes in *Irf8* ^−/−^ BM was significantly higher than that in WT, which resulted in decreased proportions of other cells (Ly‐6G^−^ and Ly‐6G^low^ cells, abbreviated as Ly‐6G^−&low^, including lymphocyte and other immune cells) for single‐cell RNA sequencing. Based on distinct expression profiles, Ly‐6G^−&low^ cells were clustered into 13 major subpopulations (**Figure** **6a**; and Figure S5b, Supporting Information). In primary CpG‐responsive cells, the ratio of *Csf1r* ^+^ macrophages/ monocytes, *Siglech* ^+^ DCs, *Cd19* ^+^ B cells were dramatically decreased in *Irf8* ^−/−^ Ly‐6G^−&low^ cells. In addition, IRF8 deficiency led to a decrease in basophilic cells and erythrocytes (Figure 6a‐b). These results suggested that the impaired proinflammatory cytokine production observed in *Irf8* ^−/−^ mice after CpG stimulation was partially attributable to a sharp decrease in the abundance of the main secretors.

IRF8 deficiency altered the phenotype and gene‐expression profile of most of the primary CpG‐responsive cells. Although in pDCs IRF8 deficiency can lead to decreased type I interferon production under CpG treatment,^[^ ^11^, ^22^ ^]^ it still remains unclear whether IRF8 plays a role in TLR9 signaling in other immune cells. In our research, purified spleen derived *Irf8* ^−^ *^/^* ^−^ CD19^+^ B cells produced similar levels of IL‐6 and IL‐10 to that of WT CD19^+^ B cells after CpG treatment (Figure S6a,b, Supporting Information), while they lost the ability to produce TNF*α* under stimulation with CpG (Figure S6c, Supporting Information).

To assess the impacts of IRF8 deficiency on response to CpG by cDCs and macrophages/monocytes, we measured intracellular IL‐1*β* levels in CD11b^+^MHCII^+^CD11c^+^ cDCs, CD11b^+^Ly‐6G^−^Ly‐6C^−^F4/80^+^macrophages, and CD11b^+^Ly‐6G^−^Ly‐6C^hi^ monocytes at 3 h after CpG stimulation in vivo (Figure 6c; and Figure S6d,e, Supporting Information). Compared with the PBS group, all three of these subpopulations from WT BM and spleen exhibited a significant increase in the percentage of IL‐1*β* ^+^ cells, while cDCs and macrophages from *Irf8^−^* ^/^ *^−^* BM failed to increase IL‐1*β* levels (Figure 6d–f). Unlike BM cells, *Irf8^−^* ^/^ *^−^* splenic cDCs showed a slight increase and macrophages exhibited a remarkable improvement in IL‐1*β* level (Figure 6d–f). These results suggested that, to some extent, IRF8 deficiency impaired TLR9 signaling in BM immune cells that depend on IRF8 for development but did not affect TLR9 signaling in splenic macrophages.

To further assess the role of IRF8 in TLR9 signaling in macrophages, WT and *Irf8^−/−^* bone marrow‐derived macrophages (BMDMs) were cultured in vitro following the methods of a previous report (Figure S6f, Supporting Information).^[^ ^26^ ^]^ These BMDMs were then cocultured with purified WT or *Irf8^−/−^* LKs under CpG stimulation. Similar to WT macrophages, in the presence of CpG, *Irf8^−/−^* macrophages significantly increased Sca‐1 expression levels in LKs of both WT and *Irf8^−/−^* mice (Figure S6g, Supporting Information). Consistent with these results, significant amounts of type I interferons, TNF*α* and IL‐1*β*, but not type II interferons were secreted by both of WT and *Irf8^−/−^* macrophages under CpG treatment (Figure 6g,h).

Collectively, these results suggested that, in WT mice, macrophages could activate HSCs through secretion of type I interferons and other proinflammatory cytokines. Moreover, the deletion of IRF8 does not appear to affect TLR9 signaling in mature macrophages.

### 2.8 NK Cells from *Irf8^−^* ^/^ *^−^* Mice are Nonresponsive to CpG Stimulation

As the principal regulator of HSCs both during homeostasis and under infection stress, IFN*γ* can strongly promote HSCs proliferation^[^ ^8^ ^]^ and increase the proportion of Sca‐1‐positive cells in WT and *Irf8^−/−^* LKs ex vivo (Figure 3b). When challenged with CpG, IFN‐*γ* levels were significantly increased in WT but not in *Irf8^−/−^* bone marrow cavity, which represents an important factor for HSC activation (Figure 5b). However the main CpG‐responsive cells (e.g., pDCs and macrophages) secrete type I interferons, not IFN‐*γ* (Figure 6g,h).^[^ ^22^ ^]^ Previous studies and our results here showed that T and NK cells are primary sources of IFN‐*γ* production (Figure S7a, Supporting Information),^[^ ^27^, ^28^ ^]^ but whether these two cell types are the main source of IFN‐*γ* in response to CpG remains unknown. To this end, we measured the proportion of IFN*γ* ^+^ in CD3*ε* ^+^ T cells in the bone marrow and spleen 3 h after CpG or PBS challenge in vivo (Figure S7b, Supporting Information), but observed no changes compared to the PBS control group (Figure S7c,d, Supporting Information). In addition, purified spleen‐derived CD3*ε* ^+^ T cells were stimulated with CpG‐A or CpG‐B ex vivo, which revealed that neither WT nor *Irf8^−/−^* T cells secreted any IFN*γ* by 24 h after treatment with either type of CpG (Figure S7e,f, Supporting Information). However, under stimulation with CpG‐B, low levels of TNF‐*α* and IL‐6 were produced in WT T cells but not in *Irf8^−^* ^/^ *^−^* T cells (Figure S7g,h, Supporting Information). These results suggested that T cells produced negligible quantities of IFN‐*γ* under stimulation with CpG in vivo and ex vivo.

**Figure 7:**
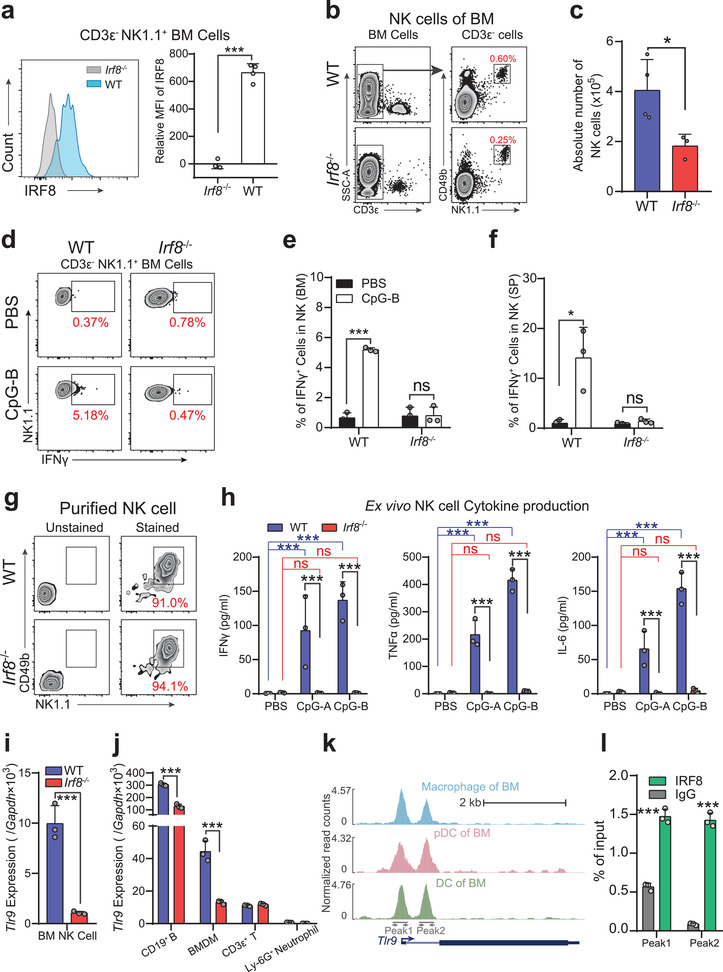
IRF8 affects the release of IFN‐*γ* from NK cells under stimulation with CpG by regulating TLR9 expression. a) The expression of IRF8 in BM NK cells was assessed by flow cytometry. b) Representative flow cytometry plots showing the frequency of BM NK cells in WT and *Irf8* ^−^ *^/^* ^−^ mice, and c) absolute counts of BM NK cells per mouse (two femurs and two tibiae). d) Flow cytometry measurements of IFN‐*γ* release by NK cells under CpG‐B stimulation. Histograms showing statistical comparisons of the percentage of IFN‐*γ* ^+^ cells in BM NK e) and spleen NK cells f). g) Representative flow cytometry plots showing purification of NK cells from BM of WT and *Irf8* ^−^ *^/^* ^−^ mice. h) Proinflammatory cytokine (IFN‐*γ*, TNF‐*α*, IL‐6) production by WT and *Irf8* ^−^ *^/^* ^−^ BM NK cells at 24 h after treatment with PBS, CpG‐A, or CpG‐B. i) The relative expression levels of *Tlr9* in NK cells isolated from WT and *Irf8* ^−^ *^/^* ^−^ BM cells. j) The relative expression levels of *Tlr9* in CD19^+^ B cells, BMDMs, CD3*ε* ^+^ T, cells and Ly‐6G^+^ neutrophils. k) Genome browser tracks showing the IRF8 binding sites in the *Tlr9* gene locus in macrophages, pDCs and DCs from BM. l) ChIP‐qPCR verified the IRF8 binding sites in NK cells. Error bars, mean ± s.e.m. ns, no significance, *P < 0.05, ***P < 0.001, data representing two or more independent experiments were analyzed with unpaired Student's *t*‐test.

Recent studies have shown that biallelic mutations in IRF8 result in impaired human NK cell maturation and function.^[^ ^29^ ^]^ In separate research, IRF8 was found to be required for NK‐cell‐mediated protection against murine cytomegalovirus (MCMV) and the development related genes expression was not different between WT and IRF8‐deficient NK cells.^[^ ^30^ ^]^ IRF8 was highly expressed in NK cells (**Figure** **7a**), and consistent with a previous study, IRF8‐deficient NK cells expressed normal CD49b and NK1.1 (Figure 7b), two mature surface markers of NK cells. However, IRF8‐deficient mice, had a decreased proportion and total counts of mature NK cells in BM (Figure 7c), mainly due to elevated neutrophil abundance in *Irf8^−/−^* bone marrow cavity. Functionally, at 3 h following CpG challenge in vivo, the ratios of IFN‐*γ* ^+^ cells were clearly elevated in WT BM and spleen NK cells compared with PBS control mice (Figure 7d–f). In contrast, CpG challenge could not induce IFN‐*γ* production in IRF8‐deficient NK cells from both BM and spleen (Figure 7e,f).

To investigate whether IRF8 is required in a cell‐intrinsic manner for TLR9 signaling of NK cells, purified NK1.1^+^ NK cells from WT and *Irf8^−^* ^/^ *^−^* BM were stimulated with CpG ex vivo (Figure 7g). By 24 h, high levels of IFN‐*γ*, TNF‐*α*, and IL‐6 (as well as a small quantity of type I interferons, IL‐1*β* and IL‐12) were detected in culture supernatants of purified WT NK cells, while *Irf8^−^* ^/^ *^−^* BM‐derived NK cells did not produce any IFN‐*γ*, nor any other detectable proinflammatory cytokines (Figure 7h; and Figure S7i, Supporting Information). This result implied that NK cells could be activated directly via TLR9, however, deficiency of IRF8 impaired this signaling.

### 2.9 IRF8 Directly Regulates the Expression of *Tlr9*

Further analysis of the single‐cell RNA‐seq data showed that the expression of *Tlr9* was dramatically down‐regulated in IRF8‐deficient Ly‐6G^−&low^ BM cells, but not other subfamily members (i.e., *Tlr7* and *Tlr8*) (Figure S7j, Supporting Information). In particular, real‐time quantitative polymerase chain reaction (qPCR) showed that loss of IRF8 led to down‐regulation of *Tlr9* transcription in NK cells by as much as 10‐fold (Figure 7i). In addition, Western blotting (WB) verified these results at the protein level (Figure S7k, Supporting Information). Moreover, in IRF8‐deficient mice, *Tlr9* was significantly reduced in primary *Tlr9*‐expressing cells, such as B cells and BMDMs (Figure 7j; and Figure S7k, Supporting Information).

Through close scrutiny of the *Tlr9* promoter region sequence, we found an EtS‐IRF composite element (EICE motif). It has been shown that IRF8 can indeed bind to the promoter region of *Tlr9* in BMDMs, where IRF8 thus regulates the basal expression of *Tlr9*.^[^ ^31^ ^]^ By searching the chromatin immunoprecipitation sequencing (ChIP‐seq) public database at Cistrome, we also found that IRF8 can potentially bind two sites in the *Tlr9* promoter region in macrophages, pDCs and DCs (Figure 7k). To investigate whether IRF8 also directly binds to the *Tlr9* promoter in NK cells, we performed chromatin immunoprecipitation coupled with quantitative PCR assay (ChIP‐qPCR) assays using the IRF8 antibody in NK1.1^+^ cells purified from WT BM. The results showed a significant accumulation of IRF8 occupancy of the *Tlr9* promoter region (Figure 7l). Collectively, our results showed that *Tlr9* is regulated directly by IRF8 in NK cells.

## 3 Discussion

IRF8 is a pivotal transcription factor in the hematopoietic system and its function is mainly reflected in two aspects, including its roles in lympho‐myelopoiesis and immune response.^[^ ^21^ ^]^ IRF8 plays central roles in the developmental regulation of myeloid cells and fate determination of some immune cells. Additive effects of the deficiency and dysfunction of immune cells in IRF8 deletion mice result in susceptibility to infection. In this study, we showed that while *Irf8^−/−^* HSCs were substantially less abundant, the capacity for repopulation by individual HSCs was enhanced. Further, we determined that the TLR9 signaling pathway was severely impaired in *Irf8^−/−^* innate immune cells. In agreement with the elevated repopulation capacity of IRF8‐deficient HSCs observed here, previous studies showed that impairment of inflammatory signaling pathways, including the deletion of *Ifnar* (interferon alpha and beta receptor), *Ifngr1* (interferon gamma receptor 1), *Tlr4*, and *Tlr9* increased the repopulation and self‐renewal capacity of HSCs.^[^ ^8^, ^9^, ^32^ ^]^ Numerous reports have noted that long‐term stimulation with pro‐inflammatory cytokines causes functional exhaustion of HSCs.^[^ ^6^, ^8^, ^9^, ^33^, ^34^, ^35^ ^]^ Moreover, continuously activated TLR2 or TLR4 signaling increase the number of HSCs. In our research, long‐term activation of TLR9 signaling increased the abundance of HSCs, accompanied by a decreased capacity of colony formation. Collectively, we established a connection between innate immunity and hematopoietic regulation through IRF8 and the TLR9 signaling pathway.

Earlier studies and recent advances have demonstrated that HSCs could be directly stimulated by pro‐inflammatory cytokines like IFNs as well as some kinds of TLR agonists, such as LPS.^[^ ^6^, ^8^, ^9^ ^]^ Here, we confirmed that, unlike TLR2 and TLR4 agonists, the response to CpG by HSPCs is indirect, and specifically, HSPCs were activated by proinflammatory cytokines secreted by multiple immune cell types under CpG stimulation. Generally, under stimuli, TLR9 activates downstream signaling pathways and lead to production of inflammatory cytokines in immune effector cells.^[^ ^25^, ^26^, ^27^, ^28^, ^29^, ^30^, ^31^, ^32^, ^33^, ^34^, ^35^, ^36^, ^37^ ^]^ However, we found that levels of type I, type II interferon, TNF‐*α*, IL‐1*β*, and IL‐6 in bone marrow cavity of *Irf8^−/−^* mice changed little following exposure to CpG. This observation is consistent with a previous report wherein IFN*α* was undetectable in serum following CpG stimulation in *Irf8^−/−^* mice.^[^ ^37^ ^]^ Nevertheless, loss of IRF8 had no effect on the LK response to IFN‐*γ* or other pro‐inflammatory cytokines (including IFN‐*α*, TNF‐*α*, IL‐1*β*, and IL‐6), which was consistent with the low expression levels of IRF8 in HSPCs. These findings suggested that the absence of proinflammatory cytokines after CpG stimulation was the primary reason underlying the impaired response by HSPCs in *Irf8^−^* ^/^ *^−^* mouse.

In *Irf8^−^* ^/^ *^−^* mice, the absence and functional impairment of pDC were considered to be the primary reasons for impaired production of IFN‐*α*.^[^ ^37^ ^]^ In our study, other IFN*α* producers (i.e., macrophages and cDCs in BM) showed completely abolished TLR9 signaling, while splenic cDCs exhibited partial blockage of this pathway. However, splenic macrophages and BMDMs showed normal CpG responses, independent of IRF8 deficiency. It is also worth noting that these primary CpG‐responsive cells (including macrophages/monocytes, pDCs and cDCs) are IRF8‐dependent during development. So we thus infer that in *Irf8^−^* ^/^ *^−^* mice, a developmental defect is responsible for impaired TLR9 signaling and proinflammatory production following CpG stimulation in vivo.

It has been demonstrated that the response of B cells to CpG is largely unaffected by IRF8‐deficiency in NZB (New Zealand Black) mice.^[^ ^37^ ^]^ We found that production of IL‐6 and IL‐10 by *Irf8^−/−^* B cells were comparable to that of WT. However, reduced secretion of TNF‐*α* in IRF8‐deleted B cells determined the partial TLR9 signaling defect.

Previous studies showed that pDCs and cDCs mediated the activation of NK cell antiviral function by releasing type I interferons and IL‐12 under MCMV infection.^[^ ^38^, ^39^ ^]^ *Irf8^−^* ^/^ *^−^* mice produce only negligible levels proinflammatory cytokines in bone marrow cavity after CpG stimulation, which can partially, but not entirely, explain the failed activation of *Irf8^−^* ^/^ *^−^* NK cells. In our study, purified NK cells from WT bone marrow were activated by CpG directly, consistent with research by Moriyama and his colleagues’, in which murine NK cells were directly activated via TLR9 after Baculovirus infection.^[^ ^40^ ^]^ However, in our experiments, *Irf8^−^* ^/^ *^−^* NK cells could not be activated by CpG. Combined with previous reports in which IRF8 was required for NK cell‐mediated protection against MCMV^[^ ^30^ ^]^ and TLR9‐mediated the response by pDC and cDC to MCMV infection, we can reasonably hypothesize that, in NK cells, IRF8 plays an essential role in TLR9 signaling and the defense against MCMV. Collectively, NK cells can be activated by two modes following CpG stimulation in vivo: directly via TLR9 or indirectly with the assistance of type I interferons and IL‐12. However, both ways are ablated in *Irf8^−^* ^/^ *^−^* mice.

A previous report^[^ ^31^ ^]^ and multiple ChIP‐seq data from Cistrome database (http://gnosis.cistrome.org/#) have shown that IRF8 constitutively binds two conserved regions of *Tlr9* promoter in macrophages, pDCs and DCs. In our results, IRF8 tightly regulates the expression of the *Tlr9* via direct promoter binding in NK cells, and in the same regions as that of other lineages. Compared with that in B cells and BMDMs, the basal expression of *Tlr9* in NK cells is low. Loss of IRF8 leads to a nearly ten‐fold decrease in the expression of *Tlr9* in NK cells, which is the primary cause for ablation of *Tlr9* signaling.

In addition to hematopoietic cells, a variety of non‐hematopoietic cell types residing in the bone marrow cavity including endothelial cells^[^ ^41^ ^]^ and mesenchymal stromal cells have been shown to express TLRs and to participate in sensing pathogens upon infection and inflammation.^[^ ^42^, ^43^, ^44^ ^]^ In our research, since proinflammatory cytokines showed little or no increase in IRF8 deficient bone marrow cavity after CpG challenge, we therefore deduced that IRF8 might have a pivotal role in TLR9 signaling in non‐hematopoietic cell types, and the precise mechanism will be explored in the following work.

Our work highlights the effects of IRF8 on HSCs. Briefly, IRF8 affects the release of pro‐inflammatory cytokines by controlling the development of immune cells and TLR9 expression in NK cells, through which the abundance and function of HSCs are indirectly regulated. Our work clarified another function of IRF8 in the hematopoietic system in addition to hematopoietic differentiation and immune cell development, and also emphasized the dual role of pro‐inflammatory cytokines on HSCs: ① Promoting the proliferation and hematopoietic differentiation of HSCs to quickly eliminate pathogens; ② Excessive exposure or long‐term stimulation leads to HSC exhaustion, ineffective hematopoiesis, and even aging. As regulators of the release of proinflammatory cytokines, the role of IRF8 and TLR9 in the hematopoietic system needs to be further investigated under different conditions in future studies.

## 4 Experimental Section

### Mice

*Irf8^−/−^* mice were bred^[^ ^45^ ^]^ and genotyped as previously described.^[^ ^46^ ^]^ CD45.1 mice were obtained from Jackson Laboratory. CD45.1/.2 mice were generated by intercrossing C57BL/6 (CD45.2) with CD45.1 mice. All animals used in the experiments were handled following the guideline of Shanghai Jiao‐Tong University Committee for Experimental Animals. The animals were kept in individually ventilated cages with filtered germ‐free air and maintained with sterilized water and irradiation food.

### Cell Staining for FACS Analysis and Sorting

All antibodies (Abs) for flow cytometry were purchased from Biolegend (San Diego, CA) and BD Biosciences (San Jose, CA). BM cells were harvested from femurs and tibias of 4–6‐week‐old WT and *Irf8^−^* ^/^ *^−^* mice by flushing with DPBS (Dulbecco's phosphate‐buffered saline) using 26 G needle and syringe, and then were incubated with the following biotinylated antibodies against lineage markers: B220 (RA3‐6B2), CD3*ε* (17A2), Ter‐119 (TER‐119), CD11b (M1/70), Gr‐1 (RB6‐8C5). For HSC analyzing cells were stained with fluorochrome conjugated streptavidin, followed by incubating with fluorochrome labeled antibodies against c‐kit (2B8), Sca‐1 (D7), CD150 (TC15‐12F12.2), CD48 (HM48‐1), CD34 (SA376A4), CD135 (A2F10). Raw data were analyzed with FlowJo software (Tree Star). For HSC (LSK, CD48^−^CD150^+^) sorting, total BM cells were stained with streptavidin conjugated magnetic beads (Biolegend) to deplete lineage positive cells. The lineage depleted cells were then stained with antibodies against c‐Kit, Sca‐1, CD48, and CD150 and were sorted on a FACS Aria III (BD Biosciences).

For peripheral blood analysis, 20 *μ*L of PB were lysed with red blood cell lysis buffer and fluorochrome conjugated antibodies against CD45.1 (A20), CD45.2 (104), CD11b (M1/70), CD19 (6D5), CD3*ε* (17A2) were used for staining.

### In Vivo TLR Agonists Treatment and BrdU Labeling

BrdU incorporation in LT‐HSCs from CpG‐A/‐B or poly(I:C) injected mice: intravenous injection of CpG‐A or CpG‐B (10mg per mouse) with 25 *μ*L of DOTAP (dioleoyl trimethylammonium propane methylsulfate, Sigma) 24 h before analysis; intraperitoneally injected Poly(I:C) (InvivoGen) two times 72 and 24 h before analysis. All the mice above were intraperitoneally injected BrdU (1 mg per 10g body weight, ABCONE, Shanghai, China) 16 h before analysis. For BrdU labeling, BM cells were harvested from femurs and tibiae and stained with streptavidin conjugated magnetic beads (Biolegend) to deplete lineage positive cells and then stained with antibodies against surface markers above. Cells were fixed and permeabilized for 30 min at room temperature by adding 300 µL of BD Cytofix/Cytoperm buffer (BD Biosciences) and incubating with 100 µL of BD Cytoperm Permeabilization Buffer Plus (BD Biosciences) for 10 min on ice. Cells were then re‐fixed with 100 µL BD Cytofix/Cytoperm buffer for 5 min on ice, incubated with 100 µL of DNase1 (deoxyribonuclease 1, 5 mg mL^−1^, Sangon Biotech) at 37 °C for 1 h and intracellular stained with fluorochrome conjugated antibodies against BrdU for 30 min at room temperature.

### Bone Marrow Reconstitution Experiments

In noncompetitive HSC transplantation experiments, WT and *Irf8^−/−^* LT‐HSCs (LSK, CD48^−^CD150^+^) were sorted and 150 WT or *Irf8^−/−^* HSCs were transplanted into lethally irradiated (900 cGy) congenic CD45.1 recipient. In secondary transplants, 1 × 10^6^ BM cells from primary recipients were transplanted into lethally irradiated (900 cGy) secondary recipients. In competitive transplantation, purified CD45.2 WT HSCs or *Irf8^−/−^* HSCs were respectively mixed with the same amount of purified CD45.1 WT HSCs and 200 mixed HSCs were transferred into lethally irradiated (900 cGy) congenic CD45.1/.2 recipients. All experimental procedures were performed more than 16 weeks after transplantation. Donor frequency and lineage commitment were assessed every 4 weeks by flow‐cytometric analysis of peripheral blood.

### Cell Isolation and Culture

For macrophages culture in vitro, BM cells were isolated from femurs and tibias of 4 to 6‐week‐old WT and *Irf8^−^* ^/^ *^−^* mice, and then passed through a 70 µm cell strainer to obtain single‐cell suspension. After lysed with red blood cell lysis buffer, total BM cells were centrifuged and resuspended at 10^6^ cells mL^−1^ in culture medium consisting of DMEM (Dulbecco's Modified Eagle's medium), 10% FBS (Fetal Bovine Serum), penicillin, streptomycin, amphotericin and 2‐mercaptoethanol (0.5 mM) supplemented with murine M‐CSF (50 ng mL^−1^, R&D) for 7 days. On the fourth day of culture, half of the medium was replaced by fresh complete medium. The identity of macrophage was confirmed by flow‐cytometric analysis using fluorochrome conjugated antibodies against CD11b, and F4/80 (BM8).

For NK cell isolation, BM cells were isolated into a single‐cell suspension and stained with biotinylated antibodies against NK1.1 (PK136) at 4 °C for 25 min, then incubated with Streptavidin Microbeads (Miltenyi Biotec) to deplete NK1.1 negative cells. The purity of NK cells was verified by flow‐cytometric analysis using fluorochrome conjugated antibodies against CD3*ε*, CD49b (DX5) and NK1.1. The same procedure was applied for spleen T cell and B cell isolation, except that the antibody was replaced by biotinylated antibody against CD3*ε* or CD19.

### Cytokine Detection of Bone Marrow Cavity and Cell Culture Supernatant

For cytokine analysis of bone marrow cavity, BM cells from two femurs and two tibias were flushed out and resuspended with 200 µL DPBS. After centrifugation for 5 min at 300 g, the supernatant was obtained and stored at −80 °C. For cytokine analysis of cell‐culture supernatant, 50 000 purified NK cells or 100 000 T or B cells were stimulated in 100 µL medium (for macrophage, 500 000 cells in 1 mL medium) with CpG‐A (2 µg mL^−1^) or CpG‐B (0.3 µg mL^−1^) for 24 h in a 96‐well plate (macrophages in 12‐well plates). Then, the medium was collected.

The cytokine level was measured by LEGENDplex Mouse Anti‐Virus Response Panel according to the manufacturer's instructions (Biolegend).

### CFSE Labeling and Chasing

IRF8‐deficeint LKs (Lineage*^−^*, c‐Kit^+^ cells) were sorted and labeled with CFSE according to the manufacturer's instructions (Thermo Fisher Scientific). CFSE‐labeled cells were transfused i.v. into WT recipient mice without irradiation. After 2 days, mice were treated with 200 µL of PBS or CpG‐B (10 mg per mouse) with 25 µL of DOTAP and Sca‐1 expression level of CFSE^+^ LKs were analyzed on day 3 by flow cytometry.

### Ex Vivo BM Cells and LKs Stimulation

BM cells or purified LKs of *Irf8^−/−^* or WT mice were stimulated in 500 µL medium (RPMI 1640 (Roswell Park Memorial Institute 1640) with 10% FBS, penicillin, streptomycin, amphotericin and 2‐mercaptoethanol(0.5 × 10^−3^ m)) with CpG‐A (2 µg mL^−1^, InvivoGen), CpG‐B (0.3 µg mL^−1^, InvivoGen), LPS (100 ng mL^−1^, InvivoGen), IFN‐*α* (100EU mL^−1^, R&D), IFN‐*γ* (50 ng mL^−1^, PeproTech), TNF‐*α* (300 ng mL^−1^, PeproTech), IL‐1*β* (100 ng mL^−1^), or IL‐6 (100 ng mL^−1^, R&D) for 16 h in a 24‐well plate.

### In Vivo Stimulation and Detection of the Ratio of IL‐1*β* ^+^ Cells and IFN‐*γ* ^+^ Cells

WT and *Irf8* ^−^ *^/^* ^−^ mice were administered 10 µg of CpG‐B dissolved in 200 µL sterile DPBS or 200 µL DPBS only by intravenous (i.v.) injection and were sacrificed 3 h later. For cDC (CD11b^+^MHC II^+^CD11c^+^) analysis, single‐cell suspensions from BM and spleen were stained with antibodies against surface marker CD11b, MHC II, and CD11c. For macrophage/monocyte (CD11b^+^Ly‐6G^−^F4/80^+^Ly‐6C^−^/CD11b^+^Ly‐6G^−^Ly‐6C^hi^) analysis, cells were stained with antibodies against CD11b, Ly‐6G, F4/80, and Ly‐6C. For NK cell (CD3*ε* ^−^NK1.1^+^) analysis, cells were stained with antibodies against CD3*ε* and NK1.1. After incubating for 30 min on ice, cells were fixed and permeabilized for 30 min at room temperature by adding 300 µL BD Cytofix/Cytoperm buffer (BD Biosciences), followed by intracellular staining with fluorochrome conjugated antibodies against IL‐1*β* (eBioscience) or IFN*γ* (Biolegend) in 100 µL Perm/Wash (BD Biosciences) for 30 min on ice. Cells were washed with 1 mL of Perm/Wash two times and analyzed with flow cytometry.

### RNA‐seq of LT‐HSCs

LT‐HSCs (LSK, CD48^−^, CD150^+^) were sorted into 15 mL centrifuge tube containing 3 mL DPBS with 1% FBS, centrifuged for 5 min at 300 g, and ≈50 µL of supernatant was reserved. The cells were then resuspended and counted under microscope. 30–50 LT‐HSCs were obtained for mRNA amplification which was performed following the manufacturer's instructions (Vazyme, N712‐03) with 18 amplification cycles. cDNA concentration was determined by Qubit Flex Fluorometer (Thermo Fisher Scientific) and fragment size distributions were verified by Agilent Bioanalyzer 2100.

For library preparation, TruePrep DNA Library Prep Kit V2 for Illumina (Vazyme, TD503) was used following the manufacturer's instructions.

### ChIP‐seq Data Analysis and ChIP‐qPCR

IRF8 ChIP‐seq data for macrophages, pDCs and DCs were obtained from references.^[^ ^47^, ^48^, ^49^ ^]^ (GEO accession: GSE1356193, GSE1531743, GSE 1 289 235). ChIP‐seq data were processed by Cistrome analysis pipeline and were loaded in UCSC genome browsers for visualization.

Purified NK1.1^+^ cells of WT BM were harvested and crosslinked with 1% formaldehyde for 10 min at room temperature. After sonication, the soluble chromatins were incubated with IRF8 antibody (CST) and Protein A (Millipore) overnight. Chromatin immunocomplexes were then precipitated with Protein A. The immunoprecipitated complex was washed, and DNA was extracted and purified by QIAquick PCR Purification Kit (Qiagen). ChIP DNA was analyzed by qPCR using specific primers, and the data were normalized by input DNA. Primer sequences for qPCR: Peak1‐F: 5′‐CCACCTGCTCTTTCAGGGTA‐3′, Peak1‐R: 5′‐ATGTTCCACACCAGGGTCTC‐3′, Peak2‐F: 5′‐CAGGAGGCTAGTGGAGCAAG‐3′, Peak2‐R: 5′‐GGGCTCTCCCACTTTCTCTT‐3′.

### Statistical Analyses

Statistical significance was calculated with Prism 7.0 (GraphPad Software, La Jolla, CA, USA) utilizing the Student's *t*‐test for comparison between two groups of average value and one‐way ANOVA for comparison between multiple sets of means. *P*‐values and sample size (n) for each statistical analysis are detailed in figures and figure legends.

## Conflict of Interest

The authors declare no conflict of interest.

## Supporting information

Supporting Information

## Data Availability

The data that support the findings of this study are available in [GEO] at [https://www.ncbi.nlm.nih.gov/geo/], reference number [48–50]. These data were derived from the following resources available in the public domain: [https://www.ncbi.nlm.nih.gov/geo/query/acc.cgi?acc=GSM1356193ï;›https://www.ncbi.nlm.nih.gov/geo/query/acc.cgi?acc=GSM1531743ï;›https://www.ncbi.nlm.nih.gov/geo/query/acc.cgi?acc=GSM1289235]
