# Supplementary material for: IRF8 Impacts Self‐Renewal of Hematopoietic Stem Cells by Regulating TLR9 Signaling Pathway of Innate Immune Cells
Source: Adv Sci (Weinh). 2021 Aug 8;8(19):2101031. doi: 10.1002/advs.202101031 (PMC8498865; doi:10.1002/advs.202101031)
Supplement: Supplementary file 1 — Supporting Information [file ADVS-8-2101031-s001.pdf]

WILEY-VCH

© 2021 Wiley-VCH GmbH

ADVANCED SCIENCE
Open Access

Supporting Information

for *Adv. Sci.*, DOI: 10.1002/advs.202101031

IRF8 impacts self-renewal of hematopoietic stem cells by regulating TLR9 signaling pathway of innate immune cells

Donghe Li, Yuyin Zhang, Qingsong Qiu, Jinzeng Wang, Xuemei Zhao, Bo Jiao, Xiuli Zhang, Shanhe Yu, Pengfei Xu, Yuqing Dan, Xinhua Xiao, Peihong Wang, Mingzhu Liu, Zhizhou Xia, Zhangsen Huang, Ruihong Zhang, Jiaoyang Li, Xi Xie, Yan Zhang, Chenxuan Liu, Ping Liu, Ruibao Ren**

Supporting Information

Title

IRF8 impacts self-renewal of hematopoietic stem cells by regulating TLR9 signaling pathway of innate immune cells

*Author(s), and Corresponding Author(s)**

Donghe Li, Yuyin Zhang, Qingsong Qiu, Jinzeng Wang, Xuemei Zhao, Bo Jiao, Xiuli Zhang, Shanhe Yu, Xinhua Xiao, Peihong Wang, Mingzhu Liu, Zhizhou Xia, Zhangsen Huang, Ruihong Zhang, Jiaoyang Li, Pengfei Xu, Yuqing Dan, Xi Xie, Yan Zhang, Chenxuan Liu, Ping Liu, Ruibao Ren**

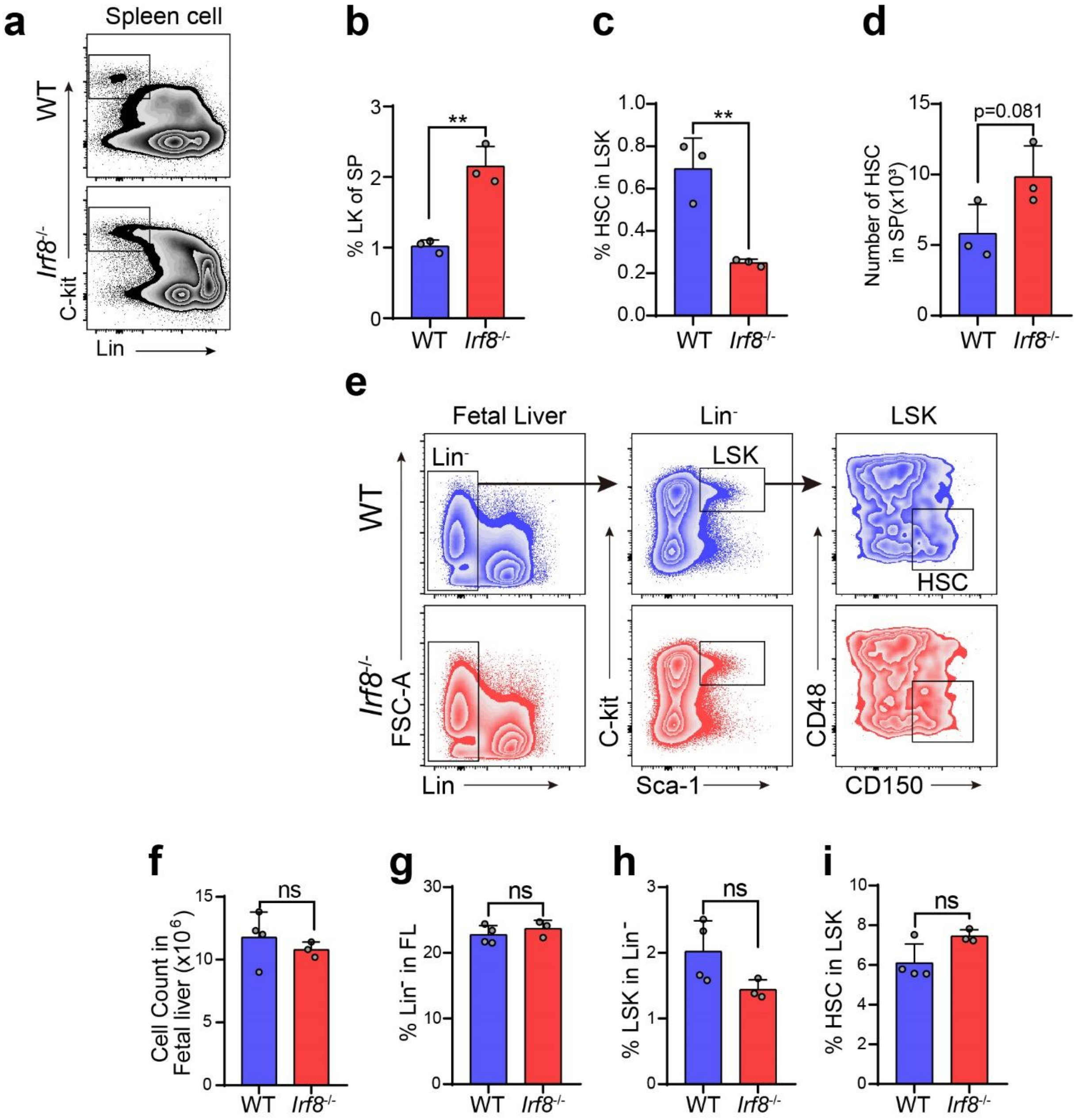

Figure S1. Loss of IRF8 increased the numbers of spleen-derived LT-HSCs from 4-week-old mice and did not change the numbers of LT-HSCs derived from E14.5 mice fetal livers. a) Representative FACS plots of LKs in spleen cells from 4-week-old WT or *Irf8*$^{-/-}$ mice. b) Proportions of LKs in spleen cells and c) the HSC proportions in LSKs. d) Absolute numbers of HSCs in spleen of 4-week-old WT or *Irf8*$^{-/-}$ mice. e) Gating strategy for the analysis of HSCs in the fetal liver cells from E14.5 embryos of WT or *Irf8*$^{-/-}$ mice. f) Total cell numbers in E14.5 fetal liver, and g) the proportions of lineage-negative cells in it. h) Proportions of LSKs in Lin^{-} cells and (i) the HSC proportions in LSKs of WT or *Irf8*$^{-/-}$ fetal livers. Error bars, mean±s.e.m. ns, not significant, ** P<0.01, data were analyzed with unpaired Student's t-test.

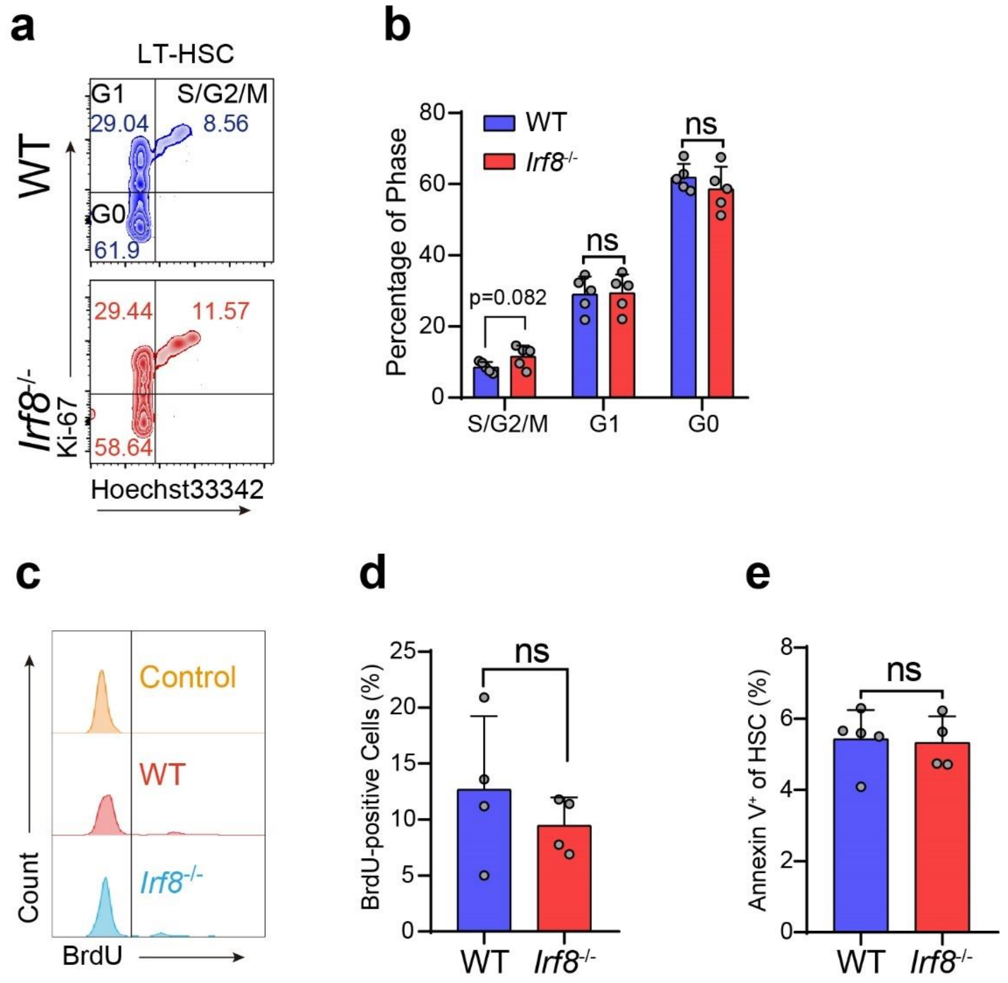

Figure S2. Loss of IRF8 did not affect the cell cycle and apoptosis of LT-HSCs. a) Representative flow-cytometric cell-cycle analysis of HSCs (LSK CD48$^-$ CD150$^+$) from 4-week-old WT or *Irf8*$^{-/-}$ mice at steady-state. G0: Hoechst33342$^-$, Ki-67$^-$; G1: Hoechst33342$^-$, Ki-67$^+$; S/G2/M: Hoechst33342$^+$, Ki-67$^+$. b) Percentage of cells in different cell-cycle phases (G0, G1, and S/G2/M) of HSCs. c) Representative FACS histograms of HSCs in BrdU incorporation assay. d) Percentage of BrdU-positive cells in HSCs from 4-week-old WT or *Irf8*$^{-/-}$ mice at steady-state, after 16 hours of BrdU incorporation. e) Apoptosis analysis of HSCs from 4-week-old WT or *Irf8*$^{-/-}$ mice at steady-state by Annexin V staining. Error bars, mean±s.e.m. ns, not significant, data were analyzed with unpaired Student's t-test.

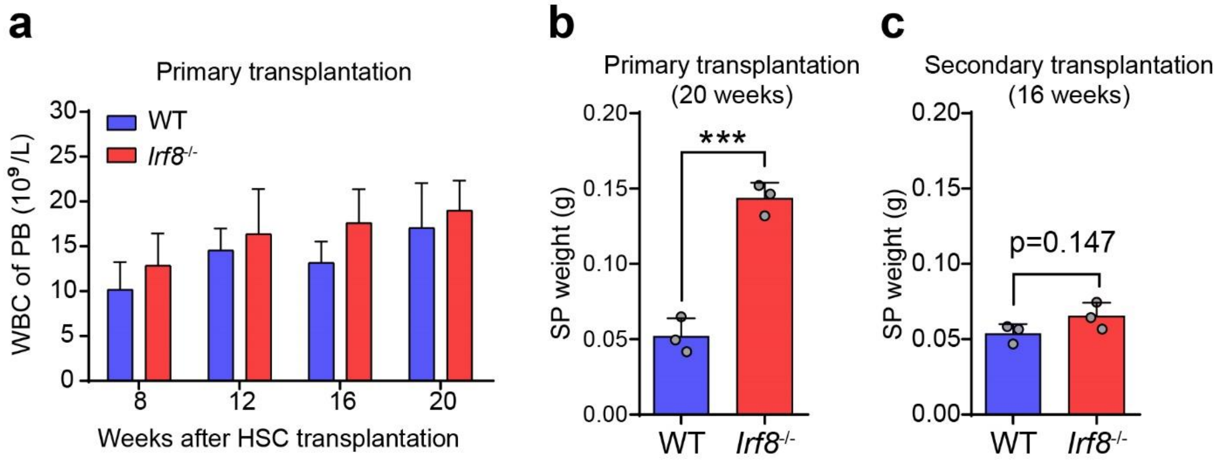

Figure S3. IRF8-deficient HSCs showed increased repopulation capacity. a) Monthly analysis of white blood cell counts in PB of primary HSC transplanted mice (WT: n=10; KO: n=10). b) Spleen weight of primary HSC transplanted mice. c) Spleen weight of secondary transplanted recipient mice.

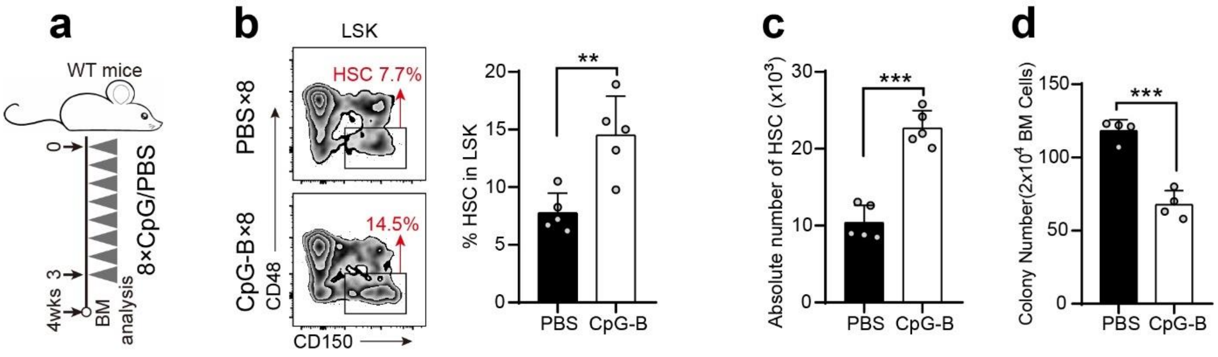

Figure S4. Long-term treatment with CpG-B increased proportions of HSCs in LSKs and absolute numbers of HSCs, while impaired the colony-forming capacity. a) Experimental scheme for long-term treatment with PBS or CpG-B. b) Representative flow plots gated on HSCs in LSKs after 8 successive doses of PBS or CpG were injected (left) and the percentages of HSCs within the LSKs (right). c) Absolute numbers of HSCs after 8 successive doses of PBS or CpG were injected. d) Colony numbers counted in the colony-forming unit (CFU) assay of total BM cells isolated from mice treated 8 times with PBS or CpG. Error bars, mean±s.e.m. ***P*<0.01, ****P*<0.001, data were analyzed with unpaired Student's t-test.

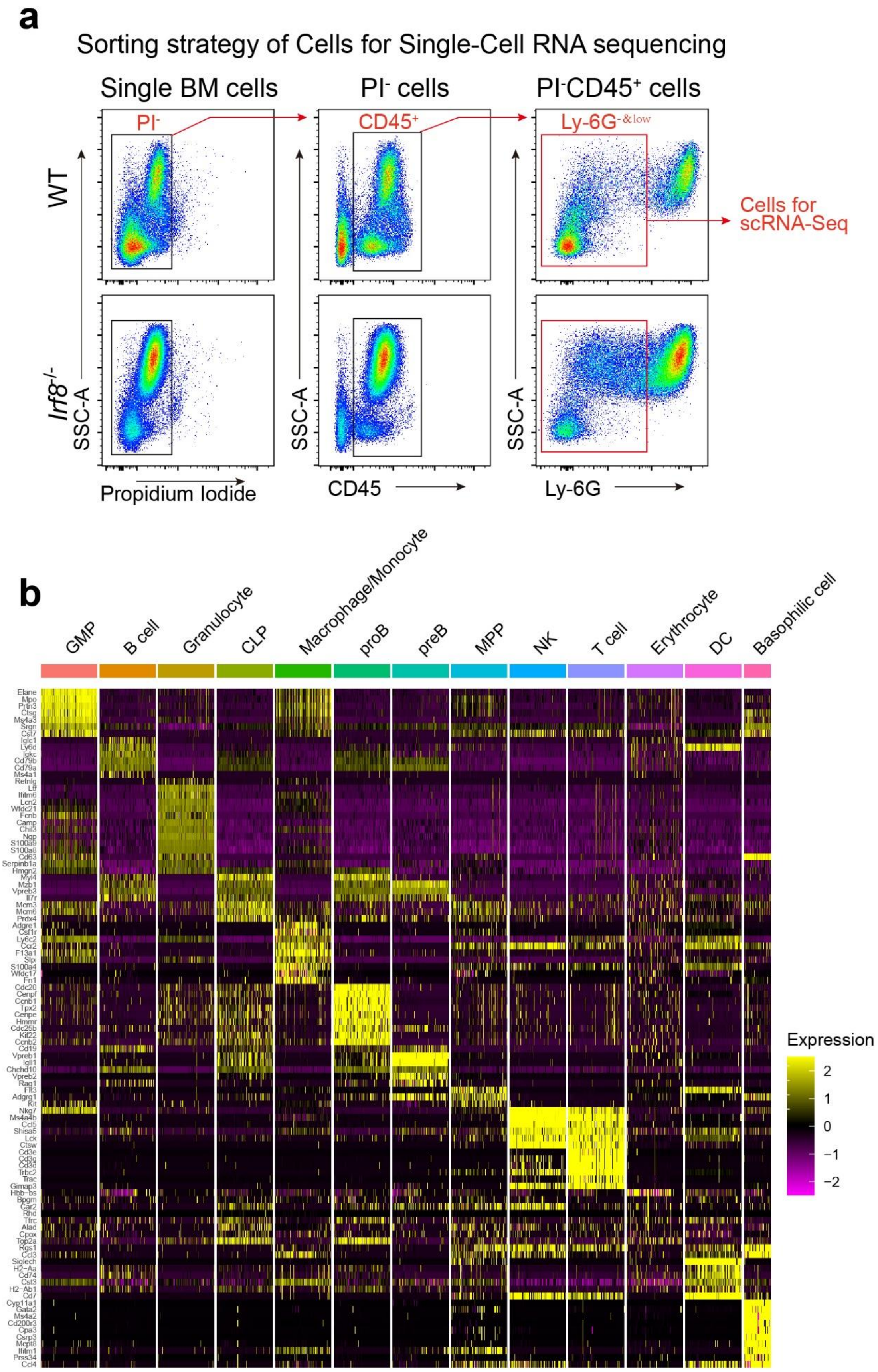
a
Sorting strategy of Cells for Single-Cell RNA sequencing
Single BM cells
PI- cells
PI-CD45+ cells
PI-
CD45+
Ly-6G-&low
WT
Irf8-/-
SSC-A
Cells for scRNA-Seq
Propidium Iodide
CD45
Ly-6G
b
GMP
B cell
Granulocyte
CLP
Macrophage/Monocyte
proB
preB
MPP
NK
T cell
Erythrocyte
DC
Basophilic cell
Expression
2
1
0
−1
−2

Figure S5. Single-cell RNA sequencing of *Irf8*$^{-/-}$ Ly-6G^{hi} depleted BM cells. a) Sorting strategy for Ly-6G$^{-\&low}$ BM cells from WT or *Irf8*$^{-/-}$ mice by FACS for single-cell RNA sequencing. b) Unsupervised hierarchical clustering of cells based on the gene expression profiles from single-cell RNA sequencing.

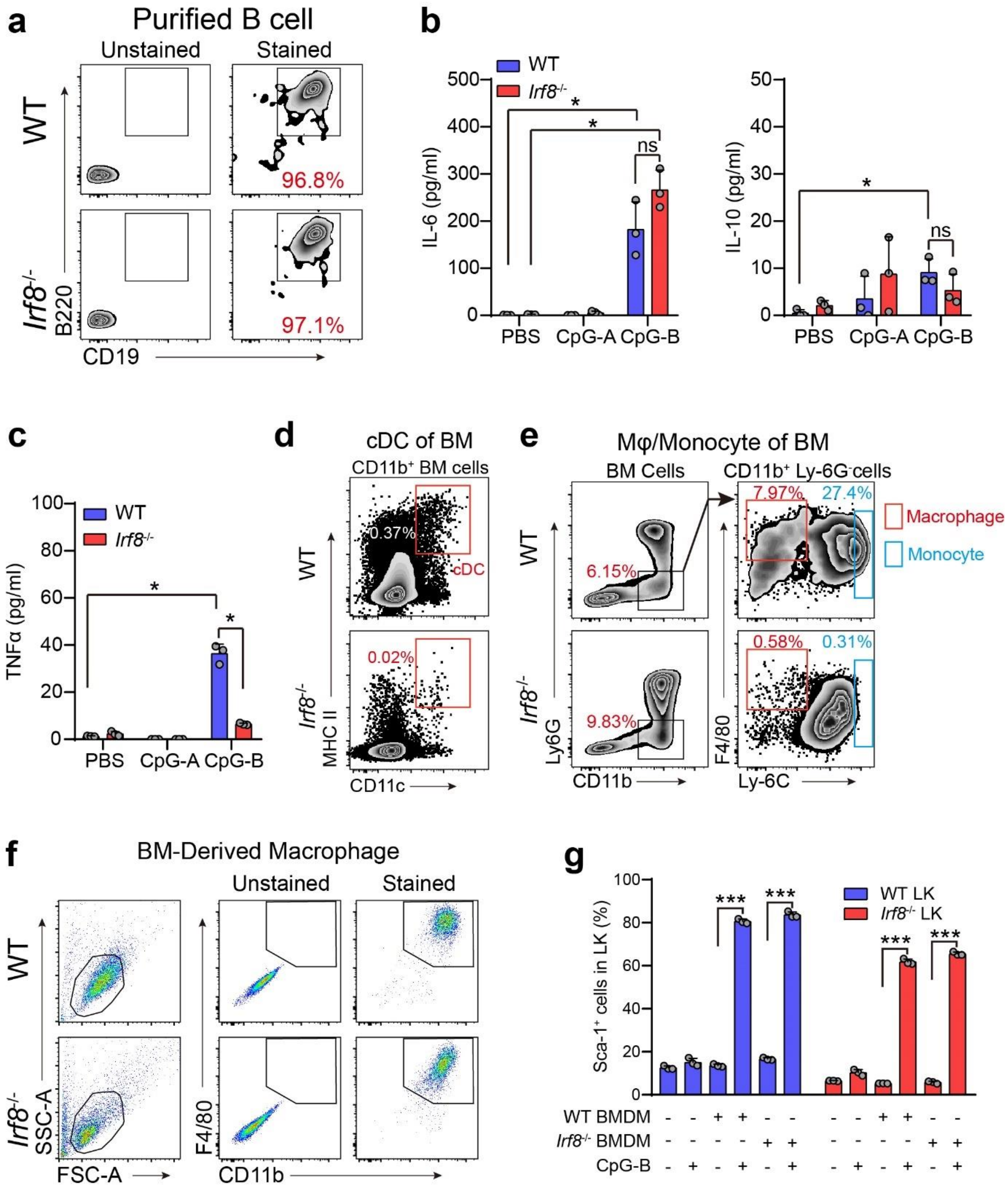

Figure S6. IRF8 is essential for TLR9 signaling in IRF8-dependent immune cells. a) The purity of B cells was verified by flow cytometry after isolation. b) The levels of IL-6, IL-10, and c) TNFα detected in purified B cell culture supernatant at 24 hours after stimulation with PBS, CpG-A or CpG-B. d) Representative flow plots gated on BM cDCs (CD11b$^+$, MHCII$^+$, CD11c$^+$). e) Representative flow plots

gated on BM monocytes (Ly-6G$^-$ CD11b$^+$ Ly-6C^{hi}) and BM macrophages (Ly-6G$^-$ CD11b$^+$ Ly-6C$^-$ F4/80$^+$). f) Flow cytometric analysis of expression of F4/80 and CD11b of BMDMs generated from WT and *Irf8*$^{-/-}$ BM. g) Proportions of Sca-1-positive cells in purified WT and *Irf8*$^{-/-}$ LKs at 16 hours after PBS or CpG-B stimulation, co-culturing with or without WT or *Irf*8$^{-/-}$ BMDMs *in vitro*. Error bars, mean±s.e.m. ns, no significant, *P<0.05, ***P<0.001, data were analyzed with unpaired Student's t-test.

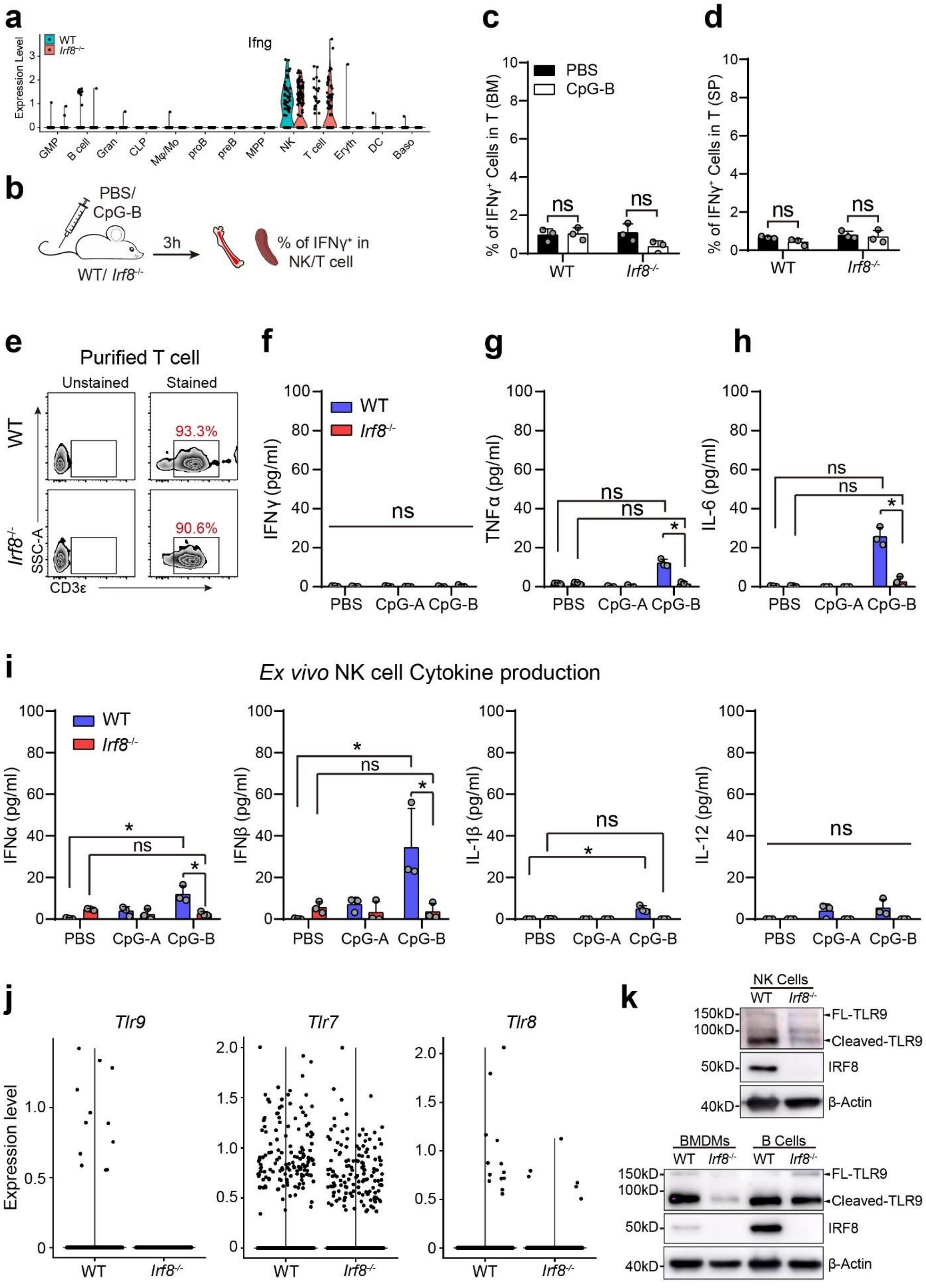

Figure S7. The TLR9 signaling pathway of BM NK cells was abolished in *Irf8*$^{-/-}$ mice. a) The *Ifng* expression level from single-cell RNA-seq data. b) Experimental scheme for measuring the

percentages of IFNγ^{+} cells in T cells and NK cells by flow cytometry at 3 hours after treating WT and *Irf8*$^{-/-}$ mice with PBS or CpG-B (i.v.). c) Percentages of IFNγ^{+} cells in BM T cells and d) spleen T cells at 3 hours after PBS and CpG-B stimulation. e) Purified T cells were verified by flow cytometry. f-h) The production of IFNγ, TNFα, and IL-6 by WT or *Irf8*$^{-/-}$ splenic T cells at 24 hours after PBS, CpG-A or CpG-B stimulation. i) IFNα, IFNβ, IL-1β and IL-12 production of WT or *Irf8*$^{-/-}$ BM NK cells at 24 hours after PBS, CpG-A or CpG-B stimulation, respectively. j) The expression level of *Tlr9*, *Tlr7* and *Tlr8* of Ly-6G$^{-\&low}$ BM cells from single-cell RNA-seq data. k) The protein levels of TLR9 were down-regulated in NK cells, BMDMs and B cells. FL-TLR9, full-length TLR9; Cleaved-TLR9, truncated and functional TLR9. Error bars, mean±s.e.m. ns, no significant; *P<0.05; data were analyzed with unpaired Student's t-test.
